# Intensity-modulated radiation therapy for early-stage breast cancer: a systematic review and meta-analysis

**DOI:** 10.1590/1516-3180.2023.0324.R1.03072024

**Published:** 2024-12-20

**Authors:** Samir Abdallah Hanna, Bruna Salani Mota, Fabio Ynoe de Moraes, Gustavo Nader Marta, Heloísa de Andrade Carvalho, Rachel Riera

**Affiliations:** I Department of Radiation Oncology, Hospital Sírio-Libanês, São Paulo (SP), Brazil.; II Department of Obstetrics and Gynecology, Instituto do Câncer do Estado de São Paulo (ICESP/HCFMUSP), São Paulo (SP), Brazil.; III Department of Oncology, Division of Radiation Oncology, Kingston General Hospital, Queen’s University, Kingston, Ontario, Canada.; IV Department of Radiation Oncology, Hospital Sírio-Libanês, São Paulo (SP), Brazil.; V Department of Radiology and Oncology, Radiotherapy Division, Hospital das Clínicas da Faculdade de Medicina (HCFMUSP), Universidade de São Paulo (USP), São Paulo (SP), Brazil.; VI Professor, Discipline of Evidence-Based Medicine, Escola Paulista de Medicina (EPM), Universidade Federal de São Paulo (Unifesp), São Paulo, SP, Brazil; Consultant, Centre of Health Technology Assessment, Hospital Sírio-Libanês, São Paulo (SP), Brazil.

**Keywords:** Intensity-modulated radiotherapy, Breast neoplasms, Adjuvant radiotherapy, Breast cancer, Breast-conservative treatment, Postoperative radiotherapy, Acute toxicity, Late toxicity

## Abstract

**BACKGROUND:**

Radiation therapy (RT) is a standard treatment for non-metastatic breast cancer and is associated with acute and late toxicities. Intensity-modulated RT (IMRT) may decrease toxicity and is convenient for patients.

**OBJECTIVES:**

To assess the efficacy and safety of IMRT in women with early stage breast cancer.

**DESIGN AND SETTING:**

Systematic review study; Multi-institutional centers.

**METHODS:**

Seven databases were searched. Randomized controlled trials (RCT) comparing IMRT with any “non-IMRT” strategies were included. Primary outcomes were local control and acute toxicity. Cochrane Handbook was use to plan and conduct the review, and PRISMA 2020 was used to report results.

**RESULTS:**

Five RCT involving 2,556 women (n = 1,283 IMRT; n = 1,274 control arm) were included. Baseline characteristics were similar between trials and arms. Local relapse-free survival rates were not different (hazard-ratio [HR] 0.62; 95%confidence interval [CI] -0.38 to 1.62; P > 0.05); however, IMRT reduced the overall acute toxicity (RR 0.69, 95%CI 0.58 to 0.82; P < 0.00001) and acute moist desquamation (risk-ratio [RR] 0.71, 95%CI 0.60 to 0.82; P < 0.00001). Lymphedema and pneumonitis rates, and survival outcomes were not affected by IMRT. The 2-year telangiectasia rate was decreased with IMRT (RR 0.66, 95%CI 0.47 to 0.93; P = 0.02); however, edema, pain, pigmentation, or fibrosis remained unaffected. IMRT did not improve cosmesis.

**CONCLUSIONS:**

IMRT improved acute toxicity and lowered telangiectasia rates, without affecting oncological and aesthetic outcomes.

**SYSTEMATIC REVIEW REGISTRATION:**

This review was registered at Cochrane Database of Systematic Reviews 2013, Issue 3. Art. No.: CD010420. https://doi.org/10.1002/14651858.CD010420.

## INTRODUCTION

Breast-conserving surgery (BCS) followed by radiation therapy (RT),^1,2^ with or without systemic therapy, is the standard treatment for early-stage breast cancer. RT has been used in patients with breast cancer who have undergone radical mastectomy or breast-conserving treatments.^3,4^

After RT, approximately 30% of women develop high-grade acute skin toxicity, which significantly affects their quality of life.^5^ The primary risk factors for acute radiation-induced toxicity include large breast size and variations in radiation doses within tissues.^6^ However, most studies have not mentioned quality assurance standards. Given that women with breast cancer often have good prognoses and long life expectancies, reducing both acute and late toxicities while maintaining the effectiveness of RT is an important strategy.

Conventional RT involves dose distribution calculations based on a single patient outline, potentially contributing to both acute and late toxicities. Advancements in radiation oncology, including three-dimensional conformal RT and intensity-modulated RT (IMRT), have improved dose distribution homogeneity. IMRT enables the modulation of radiation beam intensities in two ways:^7,8^ the field-in-field technique, and inverse planning.

IMRT in breast cancer reduces radiodermatitis^9^ and improves breast cosmesis. However, clinicians do not routinely use it due to increased costs and associated complexity.^10,11^

## OBJECTIVE

This systematic review aimed to assess the effectiveness and safety of IMRT in women with early stage breast cancer.

## METHODS

### Design and setting

This is a systematic review of randomized controlled trials (RCTs) with a protocol previously registered in the Cochrane Database.^12^ The review was conducted according to the Cochrane Handbook for Reviews of Interventions^13^ and reported following the Preferred Reporting Items for Systematic Review and Meta-Analysis (PRISMA 2020) statement.^14^

### Criteria for including studies

Study design: Only RCTs were considered.

Participants: Women with pathologically confirmed ductal carcinoma in situ or nonmetastatic invasive breast cancer (stages I, II, and III) who underwent conservative treatment, including breast surgery, axillary management, and RT of the whole breast, with or without nodal chain inclusion. Systemic therapies were allowed.

Intervention: An experimental intervention was defined as any type of IMRT, including inverse-planning IMRT using linear accelerators and field-in-field techniques and other methods.

The control group received external-beam RT without IMRT.

Hypofractionated RT was included.^15,16^ Treatments using integrated or sequential boost (electron beam, brachytherapy, or photon beam delivery) were included. Partial breast RT was excluded. **Supplementary Material** (https://t.ly/noSz6) provides information about RT treatment planning volumes.

### Outcomes

Primary outcomes:

1) Local control, defined as recurrence in the ipsilateral breast, from randomization to the development of any local recurrence during follow-up (time-to-event outcome).
2) Acute toxicity related (breast, skin, lungand heart) was considered within 3 months of RT completion. It was classified according to the scales used by the authors of each study, otherwise, we classified them according to the National Cancer Institute Common Toxicity Criteria (NCI-CTC).^17^

Secondary outcomes:

3) Overall survival, defined as the time from randomization to any-cause death during follow-up.
4) Disease-free survival, defined as the time from randomization to relapse during follow-up.
5) Late toxicity, was considered after > 6 months of RT completion. It classified according to the scales used by the authors; otherwise, we classified them as grade 3 or 4 toxic events according to the NCI-CTC.
6) Cosmesis was classified according to the scales used by authors; otherwise, we used scores from the Harvard/Radiation Therapy Oncology Group (RTOG)/National Surgical Adjuvant Breast and Bowel Project criteria.^18^
7) Quality of life was classified according to the scales used by authors or current scores (European Organisation for Research and Treatment of Cancer [EORTC] Quality of Life Questionnaire C30 and BR-23, Global Health Score, and Arm Symptoms Score).

### Search Methods and Analyses

Details of search sources and strategies are provided in **Supplementary Material** (https://t.ly/noSz6).

The risk of bias assessment was performed independently by two authors using the Cochrane Collaboration’s risk of bias tool.^19^ A third author resolved disagreements. **Appendix 1** summarizes the risk of bias and the reasons for each judgment (https://t.ly/noSz6).

The unit of analysis was the individual participant. For bilateral synchronous tumors, each treatment was individually analyzed.

The methodological and clinical heterogeneities of the included RCTs were evaluated. Statistical heterogeneity was assessed using the chi-square test (P < 0.1 indicating significance) and I² test (I^2^ > 50% indicating high inconsistency among RCTs).

Risk ratios (RR), hazard ratios (HR), and mean differences were used to estimate effects for dichotomous, time-to-event, and continuous variables, respectively, with a 95% confidence interval (95%CI).^20^ Meta-analysis was conducted using random-effects models due to expected diversity among RCTs. Review Manager 5.4 software was used for analysis.^17^

Publication bias was investigated by visually inspecting funnel plots if at least ten studies were included in a single meta-analysis.

Authors of the included studies were contacted for missing data. Missing standard deviations were calculated using reported 95%CIs and/or standard mean errors.

The certainty of evidence was evaluated using the Grading of Recommendations, Assessment, Development, and Evaluations approach.^21^ Justifications for all judgments were presented and incorporated in the summary of findings tables.

## RESULTS

After searching major databases and trial registries, 1,593 records were retrieved. After removing 380 duplicate records, 1,195 records were screened based on titles and abstracts and 1,180 were excluded. Fifteen full-text records were evaluated, resulting in the inclusion of five RCTs reported in 13 records.^22-34^

**Figure 1:**
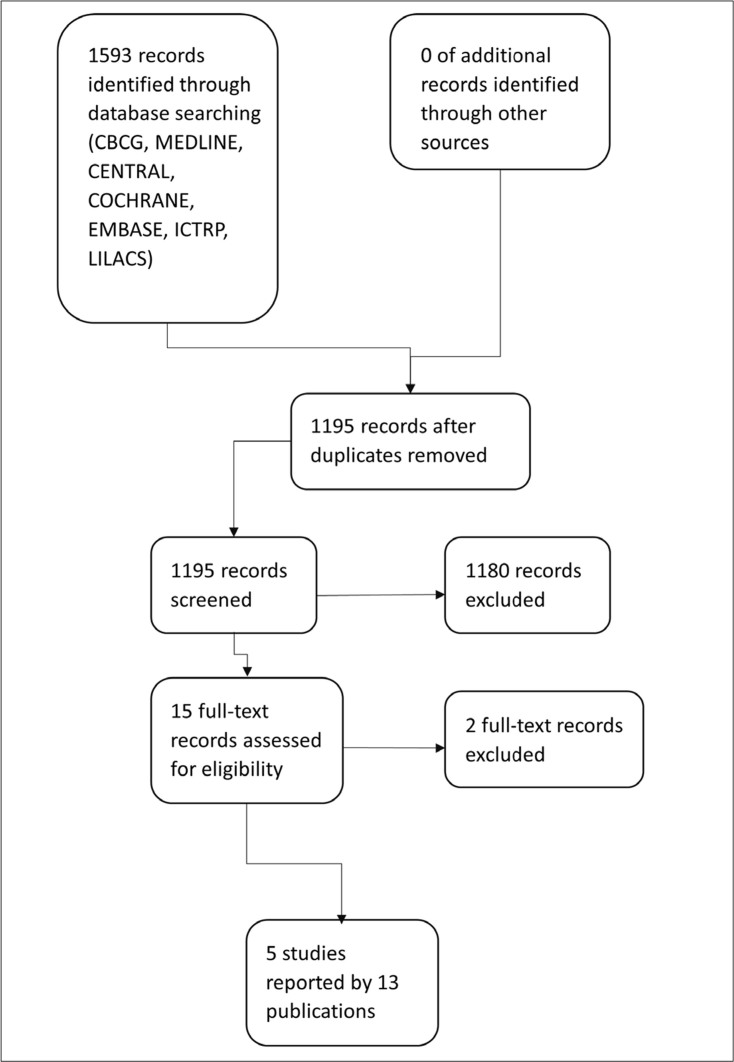
Flowchart of the Included Studies.

A flowchart of the included studies is shown in **Figure 1**. Patient characteristics are summarized in **Table 1**.

**Table 1:** Summary of Patient Characteristics in the Included Studies

Included Study	Sunnybrook Trial	Cambridge Trial	Royal Marsden Trial	MC2 Trial	KROG 15-03 Trial
RT Schedule	50Gy/25fx	40Gy/15fx	50Gy/25fx	50.4Gy/28fx + 16Gy/8fx (sequential) 50.4Gy/28fx + 64.4Gy/28fx (integrated)	50.4Gy/28fx + 9Gy/5fx (sequential) 50.4Gy/28fx + 57.4Gy/28fx (integrated)
Age (years, mean, SD/range)	57.1 (SD 10.7)	58 (34-78)	31.2 (SD 22.7)	56 (27–76)	52 (SD 9)
Smoking (n, %)	19 (11.2%)	22 (10%)	-	-	-
Chemotherapy (n, %)	52 (30.8%)	46 (20%)	63 (42%)	219 (43.6%)	442 (64%)
Hormone therapy (n, %)	67 (39.9%)	158 (73%)	126 (85%)	351 (69.9%)	505 (73%)
DM (n, %)	8 (4.7%)	9 (4%)	-	-	51 (7.5%)
Arterial hypertension (n, %)	42 (24.7%)	5 (2%)	-	-	143 (42%)
Breast size (ml, mean, SD)	585 (SD 133.8)	1,260 (285–3,436)	-	-	-
Boost (n, %)	51 (30%)	141 (62%)	150 (100%)	251 (99.2%) – IMRT arm 236 (94.7%) – 3D arm	100%
Photon energy	6 MV in 137 (80.6%)	-	6 MV/10MV	6 MV (100%)	6 MV (100%)
Mean volume V107% or V105%	V107 = 2.6%	V107 = 9.6 (0–369)	V105 = 5–10% in 6.2%	-	-
Axillary RT (n, %)	-	0	17 (11%)	72 (14.3%)	-
Supraclavicular fossa RT (n, %)	-	4 (2%)	42 (29%)	-	-

Fx = fractions; RT = radiotherapy; SD = standard deviation; DM = diabetes mellitus; MV = megavolt.

Among five RCTs, three compared IMRT with conventional RT (2D)^20,21,22^ and two^23,24^ compared IMRT with 3D-conformal RT. The following trials were included in this study.

1) Cambridge Trial (five articles):^20,26-29^ This RCT included 815 patients with, which were treated with standard RT, or re-treated with simple IMRT. Breast tissue toxicities were assessed at 5 years using photographic and clinical assessments. The groups were compared using logistic regression.
2) Royal Marsden Trial:^21^ This RCT included 306 women with early stage breast cancer treated with conventional fractionation. The primary outcome was a change in breast appearance, and the secondary outcomes were patient’s self-assessment of breast discomfort, breast hardness, quality of life and physician’s assessment of breast induration.
3) Sunnybrook Trial:^22,32^ In this RCT, the authors from two Canadian institutions included 358 women. The primary outcomes were rates of acute skin reactions and late breast pain. Secondary endpoints included breast cosmetics; quality of life; and local recurrence-free, disease-free, and overall survivals. Patients were analyzed each week during treatment and for up to 6 weeks thereafter.
4) KROG 15-03 trial:^24^ This RCT included patients from six tertiary South Korean institutions. The primary endpoint was local recurrence-free survival at 3 years. The secondary endpoints were overall, recurrence-free, and distant metastasis-free survivals; treatment-related toxicity; and dosimetric issues.
5) MC2 Trial:^23,30,31^ This German single-site RCT included 502 patients from two institutions. The primary endpoints were local control and cosmesis. The secondary endpoints were quality of life, overall survival, and disease-free survival.

**Figure 2:**
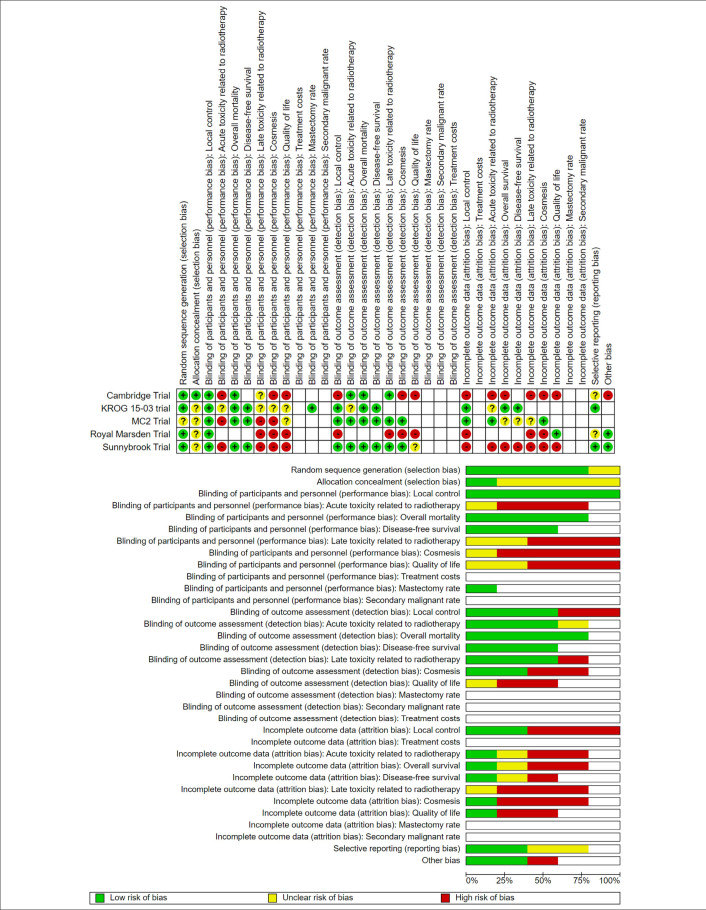
Risk of Bias Summary.

Across all five studies, 2,556 women who fulfilled the inclusion criteria were included in the IMRT arm (n = 1,283) and control group (n = 1,274). Baseline characteristics were similar between trials and radiotherapy arms. Medium-sized breasts (595 mL) were most common among the breast with known size. Smoking rate was approximately 10%. The mean volume of breast tissue receiving 107% and 105% of the prescribed dose (V107% and V105%, respectively) was low in the IMRT arm, as expected. RT to the axilla or supraclavicular fossa and boosts were included in the analysis (**Table 1**). We assessed performance and detection bias of each outcome of interest (**Supplementary Material** https://t.ly/noSz6). **Figure 2** summarizes the risk of bias after pooling the included studies.

### Primary outcomes

**Table 2** illustrates the results of the primary outcomes.

**Table 2:** Main Findings

Outcomes	Anticipated absolute effects* (95%CI)		Relative effect (95%CI)	№ of patients (studies)	Certainty of the evidence (GRADE)	Comments
	Risk with Non-IMRT	Risk with IMRT				
Local control assessed with: Risk Ratio follow-up: mean 5 years	Low		**RR 2.03** (0.85 to 4.85) [Local Control]	2249 (3 RCTs)	⊕⊕⊝⊝ Low^a,b^	
	0 per 1000	**0 per 1000** (0 to 0)				
Acute toxicity - overall (grades 3 and 4) - 2D control group assessed with: frequency of patients who experienced at least one acute toxicity	18 per 100	**11 per 100** (8 to 14)	**RR 0.61** (0.46 to 0.81)	1146 (2 RCTs)	⊕⊕⊝⊝ Low^a^	
Acute toxicity - moist desquamation assessed with: frequency of patients who experienced the event	24 per 100	**17 per 100** (14 to 20)	**RR 0.71** (0.60 to 0.83)	1824 (3 RCTs)	⊕⊝⊝⊝ Very low^a,c^	
Acute toxicity - lymphedema assessed with: frequency of patients who experienced the event	6 per 100	**4 per 100** (2 to 6)	**RR 0.62** (0.36 to 1.05)	1151 (2 RCTs)	⊕⊝⊝⊝ Very low^a,c^	
Acute toxicity - pneumonitis assessed with: frequency of patients who experienced the event	3 per 100	**2 per 100** (1 to 5)	**RR 0.65** (0.25 to 1.65)	690 (1 RCT)	⊕⊝⊝⊝ Very low^b,d^	
Overall survival follow-up: median 5 years	Low		**RR 0.32** (-0.66 to 1.30) [Death]	2006 (3 RCTs)	⊕⊕⊝⊝ Low^b,e^	
	0 per 1000	**0 per 1000** (0 to 0)			
Late toxicity - skin - Telangiectasia assessed with: frequency of patients who experienced the event follow-up: 24 months	11 per 100	**7 per 100** (5 to 10)	**RR 0.66** (0.47 to 0.93)	1374 (3 RCTs)	⊕⊝⊝⊝ Very low^a,d,e^	
Late toxicity - skin - Pigmentation assessed with: frequency of patients who experienced the event follow-up: 24 months	23 per 100	**17 per 100** (13 to 24)	**RR 0.75** (0.55 to 1.03)	633 (1 RCT)	⊕⊝⊝⊝ Very low^a,c^	
Late toxicity - skin, edema assessed with: frequency of patients who experienced the event	35 per 100	**27 per 100** (15 to 48)	**RR 0.76** (0.43 to 1.35)	1083 (2 RCTs)	⊕⊝⊝⊝ Very low^a,d,e^	
Late toxicity - breast pain assessed with: frequency of patients who experienced the event follow-up: 24 months	38 per 10	**36 per 100** (32 to 41)	**RR 0.94** (0.83 to 1.07)	1496 (3 RCTs)	⊕⊝⊝⊝ Very low^a,c^	
Late toxicity - breast induration or fibrosis assessed with: frequency of patients who experienced the event follow-up: 24 months	55 per 100	**52 per 100** (43 to 64)	**RR 0.96** (0.78 to 1.18)	1324 (3 RCTs)	⊕⊝⊝⊝ Very low^a,c,e^	
Overall cosmesis assessed with: frequency of patients with excellent or good evaluation follow-up: 24 months	86 per 100	**85 per 100** (79 to 91)	**RR 0.99** (0.92 to 1.06)	495 (1 RCT)	⊕⊕⊝⊝ Low^a^	
Quality of life follow-up: mean 5 years	The mean quality of life was 0	mean **1.45 more** (0.1 fewer to 0.12 more)		2.96 (1 RCT)	⊕⊝⊝⊝ Very low^a^	The Cambridge Trial used the EORTC QLQ BR-23 and C30 at 6, 24, and 60 months and they reported results at 2 and 5 years showing no significant differences between groups at 24 and 60 months. The Royal Marsden Trial reported no significant differences between groups at 24 and 60 months (EORTC QLQ C-30 and BR-23). The Sunnybrook Trial reported no significant differences between groups at 1 month (EORTC QLQ C-30 and BR-23). No numeric data was provided for meta-analysis.
Overall cosmesis - excellent or good evaluation - At least 5-year of follow up assessed with: frequency of patients with excellent or good evaluation follow-up: 60 months	59 per 100	**61 per 100** (56 to 66)	**RR 1.03** (0.95 to 1.11)	964 (3 RCTs)		
Overall cosmesis - excellent or good evaluation - At up to 1-year of follow-up	784 per 1000	**800 per 1000** (706 to 909)	**RR 1.02** (0.90 to 1.16)	753 (2 studies)		
Quality of life - Baseline	The mean quality of Life - Baseline was 0	MD **0.01 higher** (0.08 lower to 0.1 higher)	-	764 (1 study)	-	
Quality of life - 6 months	The mean quality of Life - 6 months was 0	MD **0.02 lower** (0.12 lower to 0.08 higher)	-	705 (1 study)	-	
Quality of life - 2 years	The mean quality of Life - 2 years was 0	MD **0.04 higher** (0.06 lower to 0.14 higher)	-	669 (1 study)	-	
Quality of life - 5 years	The mean quality of Life - 5 years was 0	MD **0.01 higher** (0.1 lower to 0.12 higher)	-	504 (1 study)	-	
Quality of life - total	The mean quality of Life - total was 0	MD **0.01 higher** (0.04 lower to 0.06 higher)	-	2642 (4 studies)	-	

*The risk in the intervention group (95% confidence interval) was based on the assumed risk in the comparison group and the relative effect of the intervention (95%CI). CI = confidence interval; MD = mean difference; RR = risk ratio; a) Unblinded patients We downgraded two levels (-2) for study limitations: b) We were not certain about patients/personnel blinding. Therefore, we downgraded CI by one level (-1): c) Wide CI, including both significant benefits and significant harm. We downgraded the CI one level (-1): d) Wide CI, including both significant benefits and harm. Low rate of events. We downgraded two levels: (-2) and e) high inconsistencies among the studies. We downgraded one level (-1).

GRADE Working Group grades of evidence: High certainty: We are very confident that the true effect lies close to that of the estimate of the effect; Moderate certainty: We are moderately confident in the effect estimate: the true effect is likely to be close to the estimate of the effect, but there is a possibility that it is substantially different; Low certainty: Our confidence in the effect estimate is limited: the true effect may be substantially different from the estimate of the effect. Very low certainty: We have very little confidence in the effect estimate; the true effect is likely to differ substantially from the effect estimate.

### Local control

Four out of five studies reported information regarding local recurrence-free survival.^20,22-24^ Recurrence events from studies comparing IMRT with 3D and conventional RT were grouped. No difference were observed among the groups (RR 1.43; 95%CI 0.71 to 2.87; I^2^ = 0%; P = 0.61; n = 2,247; 4 studies; low certainty evidence) (**Supplementary Material** https://t.ly/nUr8Z).

### Acute toxicity

All studies addressed this outcome considering the incidence of any acute toxic events (within 3 months) only in the irradiated breast, instead of the lung, heart, and other organs at risk. We grouped patients using the NCI-CTC into four categories—those with at least grade 2 skin reactions; those with moist desquamation, those with lymphedema, and those with pneumonitis.

**Figure 3:**
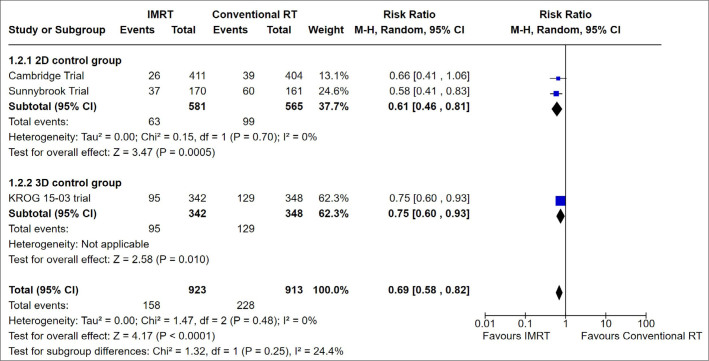
Forest Plot for Acute Overall Toxicity.

**Figure 4:**
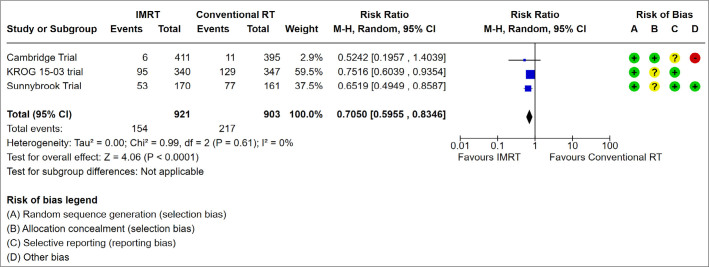
Forest Plot for Moist Desquamation.

Three RCTs^20,22,24^ reported skin reaction, two^20,22^ reported moist desquamation, two^23,24^ reported lymphedema, and one^24^ reported pneumonitis. Postoperative toxicities (wound complications, hematoma, and local infection) were not considered in these studies. The following results were obtained:

- Overall acute toxicity: lower risk with IMRT than with conventional RT (RR 0.69, 95%CI 0.58 to 0.82; I^2^ = 0%; P = 0 < 0.0001; n = 1,836; 3 studies; Low evidence certainty; **Figure 3**).
- Moist desquamation: lower risk with IMRT than with conventional RT (RR 0.71, 95%CI 0.60 to 0.83; I^2^ = 0%; P = 0 < 0.0001; n = 1,824; 3 studies; very low evidence certainty; **Figure 4**).
- Lymphedema: no difference between IMRT and conventional RT (RR 0.62, 95%CI 0.36 to 1.05, P = 0.07; I^2^ = 0%; n = 1,051; 2 studies; very low evidence certainty).
- Pneumonitis: no difference between IMRT and conventional RT (RR 0.65, 95%CI 0.25 to 1.65, P = 0.36; n = 690; 1 study; very low evidence certainty).

### Secondary outcomes

#### Overall survival

Four of the five studies reported this outcome.^20,22-24^ The HR obtained from three studies^20,23,24^ was 0.32 (95%CI -0.66 to 1.30; n = 2006; I^2^ = 0%; P = 0.52; moderate certainty of evidence).

#### Disease-free survival

Three of the five studies reported disease-free survival.^22-24^ The HR was obtained from two studies.^23,24^ Data from the Sunnybrook Trial^22^ could not be obtained because of the impossibility of calculating HRs using Parmar’s method.

The HR from two studies^23,24^ showed no significant differences in disease-free survival (HR 0.7; 95%CI -0.14 to 1.54; n = 1,192; I^2^ = 0%; P = 0.1; moderate certainty of evidence).

In the Sunnybrook Trial,^22^ no differences were observed between the groups. At 9 years, the disease-free survival rate was 82.4% for IMRT and 82% for conventional RT (P = 0.90).

#### Late toxicity

Late toxicity was evaluated in four studies^20-22,24^ as skin toxicity (telangiectasia, pigmentation changes, and breast seroma), breast pain, breast induration, or fibrosis. Toxicity to the lungs, heart, and other at-risk organs was not analyzed because of the lack of such information in the included studies.

#### Late skin toxicity (telangiectasia, edema, pigmentation change)

Three RCT assessed this outcome^20,22,24^ and the following results were found at 2 years (**Supplementary Material** https://t.ly/nUr8Z):

- Telangiectasia: lower risk with IMRT than with conventional RT (RR 0.66; 95%CI 0.47 to 0.93; n = 1,374; 3 studies; I^2^ = 0%; P = 0.02; very low certainty evidence);
- Breast edema: no difference between IMRT and conventional RT (RR 0.76, 95%CI 0.43 to 1.35; n = 1,437; 2 studies; I^2^ = 56%; P = 0.34; very low certainty evidence);
- Breast skin pigmentation: no difference between IMRT and conventional RT (RR 0.75, 95%CI 0.55 to 1.03; n = 760; 1 study; P = 0.07; very low certainty evidence).

#### Breast pain

Four RCT assessed this outcome.^20,21,24^ The Royal Marsden Trial reported no difference between the groups but did not provide the numeric data necessary to perform a meta-analysis. We performed a meta-analysis of two RCTs^20,22^ that used a four-point scale to rank outcomes.

No difference in breast pain was observed between the groups at 24 months (RR 0.94, 95%CI 0.83 to 1.07; n = 1,496; 3 studies; I^2^ = 0%; P = 0.37; very low certainty evidence) (**Supplementary Material** https://t.ly/nUr8Z).

#### Assessment of breast induration or fibrosis

Clinical breast induration was assessed in some studies.^20-22,24^ However, a pooled analysis including all studies of breast induration data could not be performed because the outcome was assessed differently in the Royal Marsden Trial.

In the Royal Marsden Trial, clinical breast induration was assessed by clinicians at different locations in the breast, and induration was observed in significantly fewer patients in the IMRT group (**Supplementary Material** https://t.ly/nUr8Z). When the author compared changes in breast appearance with clinician-reported induration, 37% of the patients who reported any change in breast appearance at 2 years also had clinician-reported induration assessed at 24 months. A similar pattern emerged at 60 months.

The meta-analysis from three studies^20,22,24^ revealed no difference in fibrosis between the groups at 24 months (RR 0.96; 95%CI 0.78 to 1.18; n = 1324; 3 studies; I^2^ = 49%; P = 0.67; very low certainty evidence).

#### Cosmesis

Four of the five clinical trials evaluated cosmesis. One study^24^ described the results using the Harvard scale^35^ and a three-point scale, two^20,21^ used a three-point scale, and one^22^ used the EORTC cosmetic breast cancer rating system.

The Cambridge Trial evaluated the photographic assessment of breast shrinkage as the primary endpoint. Frontal photographs of both breasts were taken at baseline and at 5 years post-RT. Breast cosmesis was scored on a validated three-point scale (good, moderate, or poor) by seven observers.

The MC2 Trial evaluated breast photographs as the primary endpoint. A blinded physician evaluated the photographs using a quantitative digitizer scoring system to calculate the breast retraction assessment (BRA) scores. Photographs of both breasts were taken before RT (baseline) and at 6 weeks and 2 years post-treatment. Additionally, cosmesis was assessed by treating physician and patients using the RTOG/Harvard Scale and was independently scored as excellent, good, fair, or poor. At 2-year follow-up, 378 of 502 patients (75.3%) underwent planned photographic assessments. The baseline (range) breast retraction assessment (pBRA) was 8.9% (0.5%–47.5%) for IMRT and 8.7% (0.2%–53.1%) for 3D imaging. Six weeks post-RT, cosmesis was not inferior in the IMRT group than in the control group (median [range] pBRA of 9.1% [0.7%–43.9%] vs. 9.1% [0.2%-51.2%], respectively). Non-inferiority was also detected for cosmetic outcomes 2 years post-treatment, with a median (range) pBRA of 10.4% (2.2%–32.6%) and 9.8% (0.4%–63.2%) in the intervention and control groups, respectively (95%CI 0.317% to 0.107%; P = 0.332). Cosmetic assessment according to the Harvard criteria by treating physician and patients was available 6 weeks, and 2 years post-treatment. No significant cosmetic differences were detected using these criteria between the two treatment arms at both time points of assessment

The Royal Marsden Trial evaluated breast photographs as a cosmetic outcome. Frontal images of both breasts were collected at baseline and 1, 2, and 5 years after treatment. Three observers provided scores using a 3-point graded scale (none/minimal = 0, mild = 1, marked = 2) based on the changes in breast size and shape.

The Sunnybrook Trial provided cosmetic outcomes in its second publication, with scoring based on the EORTC cosmetic breast cancer rating system during a median of 9.8 years of follow-up. The proportion of good-to-excellent cosmetic outcomes was similar between the groups (82.0% vs. 82.7% for IMRT and conventional RT, respectively). They also reported self-reported cosmesis rates in patients using the Breast Cancer Treatment Outcome Scale questionnaire.^36^

The pooled analysis from four studies^20-22,24^ revealed no difference in fibrosis between the groups at 24 months (RR 1.01, 95%CI 0.98 to 1.05; 2930 patients; 4 studies; I^2^ = 0%; P = 0.54; very low certainty evidence) (**Supplementary Material** https://t.ly/nUr8Z).

#### Quality of life

Three of the five studies described this outcome.^20,21,22^ Detailed information is available in **Supplementary Material** (https://t.ly/nUr8Z).

## DISCUSSION

This systematic review included five RCTs suggesting the benefit of reduced dose inhomogeneity for acute skin toxicity, with a 31% reduction in corresponding rates. Moreover, fewer patients presented grades ≥ 2 acute skin toxicities in the IMRT group, representing a 24% reduction. The rates of acute toxicity and local control did not differ between the groups. The overall and disease-free survival rates were similar between IMRT and conventional RT. The telangiectasia rate exhibited a 34% reduction in the IMRT group; no differences were observed in the remaining late toxicities such as breast fibrosis, breast induration, and breast pain.

This review suggests that IMRT is as effective as a non-IMRT modality concerning local control, overall survival, and disease-free survival and is less toxic to patients, regardless of other clinical features. We suppose that the reduction in acute and late toxicity outcomes has generated therapeutic benefits.

The increase in radiodermatitis rates may generate increased medical and multidisciplinary team consultations during treatment,^37^ RT interruptions owing to pain,^38^ and treatment dropout rate and reduced effectiveness. The use of hypofractionation would decrease the chance of patient withdrawal from treatment. Pertinently, the Cambridge Trial adopted moderate hypofractionation in both arms, which supports the use of IMRT even in the hypofractionation setting.

Three studies evaluated aesthetic outcomes. Our review revealed no differences between the groups.

The improvements in aesthetic outcomes observed in individual studies did not translate into significant benefits when patients were grouped for this specific endpoint, probably due to the difference in assessment, methods and timing across the studies. Furthermore, some late toxicity events (telangiectasia) may be accounted as either late toxicity or aesthetic dissatisfaction. Interpreting these data is challenging.

Regarding quality of life, pooling the data was not possible due to the lack of available data at fixed time points during follow-up. No difference was observed between the groups concerning this endpoint analyzed individually.

Cost has been analyzed in other studies besides the RCTs included in this review; however, it has not been shown to correlate with clinical endpoints. Additionally, our study group included all IMRT modalities, including simple planning with field-in-field techniques, inverse planning with IMRT of static beams and arc beams, and even concomitant boost with IMRT arm. This inclusion allowed us to conduct the proposed analyses, but the cost and complexity of simple planning are undoubtedly lower than those of inverse planning, as exemplified by a publication form the group in Louisiana.^39^ Considering this, we might infer that the use of simpler IMRT techniques would generate benefits as observed in our study. Acute toxicity is directly linked to the risk of treatment interruption,^40^ which can cause a greater demand for health services, leading to indirect effects on cost-effectiveness.

The risk of late toxicities is robustly related to the dose received by a specific tissue or organ.^40^ The radiation dose inhomogeneity within the breast has been studied as an independent predictor of acute and late toxicities, including radiodermatitis and fibrosis or breast induration. The prescribed radiation dose can vary by up to 40% within the irradiated volume. IMRT is beneficial in in (a) improving dose homogeneity, (b) sparing the contralateral breast and the lungs or heart^11^ (reducing late cardiac effects due to radiation),^41-43^ and (c) diminishing the likelihood of toxicities and secondary malignancies over time.

Radio induced cardiac and lung toxicity can manifest decades after treatment. Thus, patients with breast cancer who benefit from cancer treatment experience or even die from late toxicities, especially cardiac and pulmonary toxicities. The studies included in this review did not have sufficiently long follow-up periods to analyze the impact of IMRT on cardiac toxicity. However, previous evidence, has shown a decline in the average doses received by such organs with the use of advanced technology.^24^ The authors found a strong direct relationship between the rates of second primary cancers and cause-specific mortality and the mean doses to the lungs and heart.^44^

This review showed low overall quality of the evidence, due to the methodological quality of the RCTs. Although being randomized, most studies lacked generation of allocation sequences; allocation concealment; and blinding of participants, personnel, or outcome assessors or involved attrition bias.

The quality of evidence for the primary and secondary outcomes was considered low.

We observed some difficulty in examining late skin toxicities because many skin features occur late after treatment, and what may be considered indicators of late toxicity were often included in cosmetic analysis. Thus, we attempted to separate cosmetic analyses from toxicity itself, although this may introduce an interpretation bias.

### Study Limitations:

1. Heterogeneity in treatment protocols and endpoints across included studies;
2. Low or very low certainty of evidence for most outcomes;
3. Limited follow-up duration in some studies;
4. Limited data on quality of life.

In our review, we grouped all types of IMRT. Thus, even in centers where IMRT alone is not available, a combination of field-in-field or “modulated” tangent fields with the classical opposed tangents technique may provide the same benefits we observed.

Well-planned and well-designed studies are necessary to provide evidence about the long-term effects of IMRT and assess patient-oriented outcomes that have been poorly addressed by available studies. Further studies are needed to identify and compare all techniques, modalities, and schemes of IMRT available to date to determine the optimal option among them. Technologies are evolving and, therefore, will likely offer less toxic treatments. Finally, a prespecified subgroup analysis, including biomarkers among other variables such as baseline and dosimetric issues of patients, is desirable.

## CONCLUSIONS

In women with early stage breast cancer treated with surgery and postoperative RT, IMRT potentially reduces the risk of acute skin toxicity, specifically moist desquamation. However, no evidence suggests that IMRT improves local control, overall survival, disease-free survival, late toxicity, cosmesis, or quality of life better than conventional RT. The choice between IMRT and conventional RT should be individualized based on patient characteristics and treatment goals. Further research is needed to assess the long-term outcomes and quality of life in this population.

## References

[1] Fisher B, Anderson S, Bryant J (2002). Twenty-year follow-up of a randomized trial comparing total mastectomy, lumpectomy, and lumpectomy plus irradiation for the treatment of invasive breast cancer.. N Engl J Med.

[2] Brackstone M, Baldassarre FG, Perera FE (2021). Management of the Axilla in Early-Stage Breast Cancer: Ontario Health (Cancer Care Ontario) and ASCO Guideline. J Clin Oncol.

[3] Clarke M, Collins R, Darby S (2005). Effects of radiotherapy and of differences in the extent of surgery for early breast cancer on local recurrence and 15-year survival: an overview of the randomised trials. Lancet.

[4] Veronesi U, Cascinelli N, Mariani L (2002). Twenty-year follow-up of a randomized study comparing breast-conserving surgery with radical mastectomy for early breast cancer. N Engl J Med.

[5] Al-Ghazal SK, Fallowfield L, Blamey RW (1999). Does cosmetic outcome from treatment of primary breast cancer influence psychosocial morbidity?. Eur J Surg Oncol.

[6] Fernando IN, Ford HT, Powles TJ (1996). Factors affecting acute skin toxicity in patients having breast irradiation after conservative surgery: a prospective study of treatment practice at the Royal Marsden Hospital. Clin Oncol.

[7] Stroom JC, Heijmen BJ (2002). Geometrical uncertainties, radiotherapy planning margins, and the ICRU-62 report. Radiother Oncol.

[8] Webb S (2003). The physical basis of IMRT and inverse planning. Br J Radiol.

[9] Freedman GM, Anderson PR, Li J (2006). Intensity modulated radiation therapy (IMRT) decreases acute skin toxicity for women receiving radiation for breast cancer. Am J Clin Oncol.

[10] Smith BD, Pan IW, Shih YC (2011). Adoption of intensity-modulated radiation therapy for breast cancer in the United States. J Natl Cancer Inst.

[11] McCormick B, Hunt M (2011). Intensity-modulated radiation therapy for breast: is it for everyone?. Semin Radiat Oncol.

[12] Hanna SA., Marta GN, Riera R (2013). Intensity-modulated versus conventional radiotherapy for breast cancer. Cochrane Database Syst Rev.

[13] Higgins JPT, Thomas J, Chandler J (2019). Cochrane Handbook for Systematic Reviews of Interventions. Second Edition. Cochrane Collaboration;.

[14] Preferred Reporting Items for Systematic Reviews and Meta-Analyses (PRISMA) (2020). Reporting checklist..

[15] Haviland JS, Owen JR, Dewar JA (2013). The UK Standardisation of Breast Radiotherapy (START) trials of radiotherapy hypofractionation for treatment of early breast cancer: 10-year follow-up results of two randomised controlled trials. Lancet Oncol.

[16] Hickey BE, Lehman M (2021). Partial breast irradiation versus whole breast radiotherapy for early breast cancer. Cochrane Database Syst Rev.

[17] NCI-CTC; NCI Guidelines: Adverse Event Reporting Requirements..

[18] Harris JR, Levene MB, Svensson G, Hellman S (1979). Analysis of cosmetic results following primary radiation therapy for stages I and II carcinoma of the breast. Int J Radiat Oncol Biol Phys.

[19] Higgins JPT, Thomas J, Chandler J Cochrane Handbook for Systematic Reviews of Interventions version 6.3..

[20] Parmar MK, Torri V, Stewart L (1998). Extracting summary statistics to perform meta-analyses of the published literature for survival endpoints. Stat Med.

[21] GRADEpro GDT: GRADEpro Guideline Development Tool. (2015). McMaster University, 2015 (developed by Evidence Prime, Inc.). Version 2015. McMaster University: Evidence Prime, Inc., December 4th,. Software.

[22] Mukesh MB, Qian W, Wah Hak CC (2016). The Cambridge Breast Intensity-modulated Radiotherapy Trial: Comparison of Clinician- versus Patient-reported Outcomes. Clin Oncol.

[23] Donovan E, Bleakley N, Denholm E (2007). Randomised trial of standard 2D radiotherapy (RT) versus intensity modulated radiotherapy (IMRT) in patients prescribed breast radiotherapy. Radiother Oncol.

[24] Pignol JP, Olivotto I, Rakovitch E (2008). A multicenter randomized trial of breast intensity-modulated radiation therapy to reduce acute radiation dermatitis. J Clin Oncol.

[25] Krug D, Köder C, Häfner MF (2020). Acute toxicity of normofractionated intensity modulated radiotherapy with simultaneous integrated boost compared to three-dimensional conformal radiotherapy with sequential boost in the adjuvant treatment of breast cancer. Radiat Oncol.

[26] Choi KH, Ahn SJ, Jeong JU (2021). Postoperative radiotherapy with intensity-modulated radiation therapy versus 3-dimensional conformal radiotherapy in early breast cancer: A randomized clinical trial of KROG 15-03. Radiother Oncol.

[27] Barnett GC, Wilkinson JS, Moody AM (2012). Randomized controlled trial of forward-planned intensity modulated radiotherapy for early breast cancer: interim results at 2 years. Int J Radiat Oncol Biol Phys.

[28] Barnett GC, Wilkinson JS, Moody AM (2011). The Cambridge breast intensity-modulated radiotherapy trial: patient- and treatment-related factors that influence late toxicity. Clin Oncol.

[29] Mukesh MB, Barnett GC, Wilkinson JS (2013). Randomized controlled trial of intensity-modulated radiotherapy for early breast cancer: 5-year results confirm superior overall cosmesis. J Clin Oncol.

[30] Askoxylakis V, Jensen AD, Häfner MF (2011). Simultaneous integrated boost for adjuvant treatment of breast cancer--intensity modulated vs. conventional radiotherapy: the IMRT-MC2 trial. BMC Cancer.

[31] Mukesh MB, Qian W, Wilkinson JS (2014). Patient reported outcome measures (PROMs) following forward planned field-in field IMRT: results from the Cambridge Breast IMRT trial. Radiother Oncol.

[32] Hörner-Rieber J, Forster T, Hommertgen A (2021). Intensity Modulated Radiation Therapy (IMRT) With Simultaneously Integrated Boost Shortens Treatment Time and Is Noninferior to Conventional Radiation Therapy Followed by Sequential Boost in Adjuvant Breast Cancer Treatment: Results of a Large Randomized Phase III Trial (IMRT-MC2 Trial). Int J Radiat Oncol Biol Phys.

[33] Hörner-Rieber J, Forster T, Hommertgen A (2020). First 2-Year Results of the Multicenter, Randomized IMRT-MC2 Trial (MINT): Intensity-Modulated Radiotherapy with Simultaneous Integrated Boost versus 3-D-Conformal Radiotherapy with Consecutive Boost for Breast Cancer Patients. Int J Radiat Oncol Biol Phys.

[34] Pignol JP, Truong P, Rakovitch E (2016). Ten years results of the Canadian breast intensity modulated radiation therapy (IMRT) randomized controlled trial. Radiother Oncol.

[35] Harris JR, Levene MB, Svensson G, Hellman S (1979). Analysis of cosmetic results following primary radiation therapy for stages I and II carcinoma of the breast. Int J Radiat Oncol Biol Phys.

[36] Stanton AL, Krishnan L, Collins CA (2001). Form or function? Part 1. Subjective cosmetic and functional correlates of quality of life in women treated with breast-conserving surgical procedures and radiotherapy. Cancer.

[37] Schnur JB, Love B, Scheckner BL (2011). A systematic review of patient-rated measures of radiodermatitis in breast cancer radiotherapy. Am J Clin Oncol.

[38] Fuzissaki MA, Paiva CE, Oliveira MA (2019). The Impact of Radiodermatitis on Breast Cancer Patients’ Quality of Life During Radiotherapy: A Prospective Cohort Study. J Pain Symptom Manage.

[39] Yibo Xie, Beibei Guo, Rui Zhang (2020). Cost-effectiveness analysis of advanced radiotherapy techniques for post-mastectomy breast cancer patients. Cost Eff Resour Alloc.

[40] Taylor C, Correa C, Duane FK (2017). Estimating the Risks of Breast Cancer Radiotherapy: Evidence From Modern Radiation Doses to the Lungs and Heart and From Previous Randomized Trials. J Clin Oncol.

[41] Early Breast Cancer Trialists’ Collaborative Group (EBCTCG). (2005). Effects of chemotherapy and hormonal therapy for early breast cancer on recurrence and 15-year survival: an overview of the randomised trials. Lancet.

[42] Clarke M, Collins R, Darby S (2005). Effects of radiotherapy and of differences in the extent of surgery for early breast cancer on local recurrence and 15-year survival: an overview of the randomized trials. Lancet.

[43] Roychoudhuri R, Robinson D, Putcha V (2007). Increased cardiovascular mortality more than fifteen years after radiotherapy for breast cancer: a population-based study. BMC Cancer.

[44] Dodwell D, Taylor C, McGale P (2019). Regional lymph node irradiation in early stage breast cancer: An EBCTCG meta-analysis of 13,000 women in 14 trials. Cancer Res.

